# The power of the social: A systematic review and meta-analysis of social interventions in Global Mental Health

**DOI:** 10.1371/journal.pmen.0000625

**Published:** 2026-07-01

**Authors:** Alma Ionescu, William Byansi, Farah Sheibani, Ishrat Pabla, Abbie McDonogh, Eric Frasco, Rochelle A. Burgess

**Affiliations:** 1 Institute for Global Health, University College London, London, United Kingdom; 2 Centre for Socio-Legal Studies, University of Oxford, Oxford, United Kingdom; 3 Boston College, School of Social Work, Chestnut Hill, Massachusetts, United states of America; 4 UCL Institute for Global Health, University College London, London, United Kingdom; 5 Division of Psychiatry, Faculty of Brain Sciences, University College London, London, United Kingdom; 6 Division of Psychiatry, Faculty of Brain Sciences, University College London, London, United Kingdom; 7 Senior Research associate, University of Johannesburg, Johannesburg, South Africa; PLOS: Public Library of Science, UNITED KINGDOM OF GREAT BRITAIN AND NORTHERN IRELAND

## Abstract

Recent scholarship has emphasised the importance of social interventions in mental health, broadly defined as interventions designed to deliver social benefits that drive positive mental health consequences to their beneficiaries. While more is understood about the impact of some types (i.e., social-relational interventions) globally, impacts of social interventions addressing broader systemic and material conditions linked to mental health, such as poverty and access to welfare systems, are less understood. Addressing this evidence gap is critical for advancing a holistic agenda in the global mental health field that reflects the social realities and mental health needs of communities. We conducted a systematic review in accordance with PRISMA guidelines to advance understandings of social interventions and their role in addressing mental ill-health in low resource settings. We focused on interventions targeting conditions within the WHO MHGAP (version 2.0) guidelines, delivered in LMIC countries defined by World Bank criteria. Our protocol was registered with Prospero (CRD42022357660). Searches were conducted in October 2022 and updated in June 2025. 22,553 records were identified and independently double-screened, with 83 studies meeting inclusion criteria. We structured our discussion of findings, by level of intervention delivery: Individual and family level; Household and working life level; community level; and population level. We report on findings from 36 countries, with studies from the African continent being most represented. Meta-analysis of our most commonly explored mental health condition (depression) identified a moderate effect size (Cohen’s *d* = 0.42, P = 0.005) for social interventions, with a high level of heterogeneity. Service level interventions (primarily school-based), were most common, followed by community-level interventions. Overall, our findings indicate that the primary level of delivery did not preclude actions and impact across other levels, as many social interventions were flexible in responding directly to local needs. Implications for intervention programming in LMIC landscapes are discussed.

## Introduction

How should we best respond to the social, cultural, and political framings of mental ill-health? And how may we do so in contexts where social adversity is inextricably linked to the development and experience of mental health conditions? Since its inception, the Movement for Global Mental Health (GMH), which prioritises the reduction of inequalities in mental health services access between high and low-resource settings, has faced critique regarding its engagement with the complexities of the social landscapes shaping individual and community mental health experiences in the Global South [1–3].

The moral necessity of expanding access to basic mental health services in under-resourced settings is undeniable [4–6]. Yet to date, these efforts have primarily prioritized clinical interventions, applying task-sharing models to deliver treatments for depression [7,8], substance misuse [9] and severe mental health conditions including schizophrenia [10,11]. Implemented mainly through community-based, peer-delivered programs, such approaches have demonstrated success, with some approaches now being scaled across regions, including in Africa [12,13], South Asia [14–16], and Latin America [17,18].

Despite these successes, critiques persist regarding the limited ‘fit’ of such interventions to complex social contexts. Specifically, scholars argue that while scaling up mental health services has narrowed the treatment gap, these services often fail to sufficiently address the structural and relational realities widely recognised as drivers of poor mental health care. For example, Roberts and colleagues [19] show how tensions emerge between externally designed services and the lived realities and everyday narratives of need presented by community members. In these settings, the treatment gap as a rallying point risks oversimplifying the problem by prioritizing biomedical services over social responses. Instead, they argue that more appropriate pathways forward involve approaches that explicitly centre the social concerns shaping daily life and well-being.

In response to these critiques, recent scholarship has emphasized the importance of social interventions in the global mental health space [20]. Social interventions are broadly defined as interventions designed to deliver social benefits to their beneficiaries [21]. Burgess and colleagues [20] identify two primary orientations of social interventions for consideration in mental health: Socio-structural and Socio-relational. Socio-structurally oriented interventions address broader systemic and material conditions, such as poverty, education, and access to welfare systems. Examples increasingly present in global mental health literature include cash transfers [22], social welfare interventions [23–25]. By contrast, Socio-relational oriented interventions focus on the interpersonal and relational dimensions of well-being. These include interventions designed to reduce loneliness [26,27], befriending schemes [28,29], and parenting and peer group programs [30–32]. While socio-relational approaches are more common in high-income contexts, they are increasingly recognized as relevant to low-resource settings.

Although social interventions vary in scope, they share the common trait of being situated outside the formal health sector, and require collaboration and integration of welfare, education, and community sectors. This presents challenges but also significant opportunities in settings where health and mental health systems are underdeveloped and under resourced. By involving multiple sectors, social interventions can target the root causes of mental ill-health and complement biomedical services, as they addresses dimensions of distress rooted within the broader socio-political economy of mental health, which structure people’s everyday life and opportunities for wellbeing [33].

Moreover, there are increasing calls to incorporate social interventions into the global mental health framework and ecosystem [34,35], but a systematic synthesis of their effectiveness remains limited. Existing reviews have often focused on high-income settings or on single intervention types, such as cash transfers or social protection programs [21,36], without mapping the broader spectrum of social interventions relevant to global mental health.

This systematic review and meta-analysis aims to provide a foundation for understanding the current state of evidence in relation to social interventions in global mental health. With this as our starting point, this review explores the following broad questions:

(1) What types of social interventions exist and how are they used? (i.e., Which populations and key mental health outcomes they target?), and
(2) What is the effectiveness of social interventions in reducing poor mental health symptoms?

Addressing this evidence gap is critical for advancing a more holistic global mental health agenda and expansion of interventions that reflect the social realities of affected communities. Thus, this review will advance understandings of the role that social interventions can play in addressing mental ill-health globally, and highlight implications for research, policy, and practice, with an emphasis on LMIC settings.

### Concepts and terminology

Across contexts studied in GMH, poverty, inequality, conflict, and violence are commonplace social conditions that shape both the development and treatment of mental health conditions, as well as the broader social opportunities available to individuals and communities [37]. Conversely, our conceptual framework for this review builds on Wahlbeck and colleagues’ [38] review of interventions aimed at mitigating the effects of poverty and inequality on mental health. Table 1 outlines the different intervention levels outlined Wahlbeck and colleagues’ [38], accompanied by examples of the most common types of social interventions identified at each level. While their work is primarily focused on high-income settings, we adapt their categorisation of intervention levels to guide our search strategy, as they provide a useful starting point, with a clear articulation of the different domains at which social interventions may operate to improve the social conditions of individuals and communities in the Global South.

**Table 1:** Adaptation from Wahlbeck et al (2017) of intervention levels where social interventions may operate.

*Intervention: level of delivery*	*Examples*
Individuals, families	Individual treatment support, skills programmes, parenting programmes
Households and working life	Interventions that target household or workplace environments. Includes support for workplace interventions, family support, and employment interventions
Community level	Community and NGO services, neighbourhood support
Service level	Provision of services at healthcare settings and schools Interventions that blend therapy with social interventions
Country level	Policy level interventions that target entire populations, which may include cash transfer programmes, social protection policies, education access, grant programmes, housing services

## Methods

### Literature search

This review was conducted in accordance with the Preferred Reporting Items for Systematic Reviews and Meta-analyses (PRISMA) guidelines [39]. The protocol was prospectively registered with Prospero (CRD42022357660). No amendments were made to the search terms. Our Prisma checklist can be found in supporting information files (see S1 Checklist).

**Fig 1:**
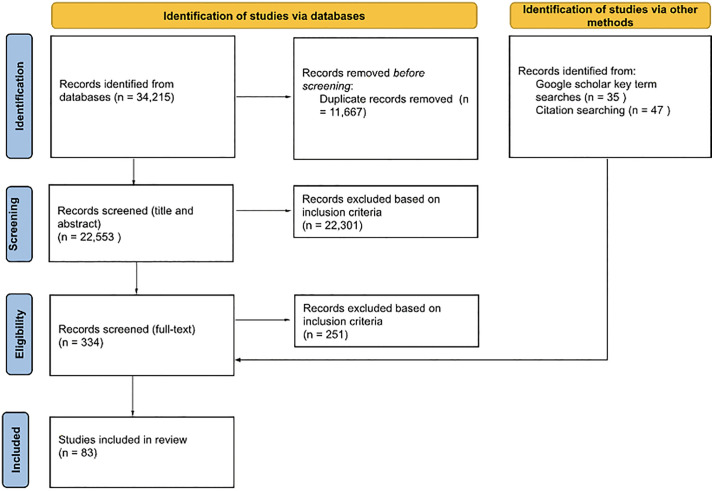
PRISMA flow chart outlining screening process.

The primary search was conducted in four major databases: Medline, PsycINFO, Embase, and Global Health. The search strategy (see Table 2) included a string of terms related to social interventions. To capture studies in low- and middle-income countries (LMICs), we applied an adaptation of the Cochrane EPOC LMIC geographical filter. The initial search was conducted in October 2022 and further updated in June 2025. In total, 22,553 records were identified and independently double-screened by a team of five trained reviewers (AI, FS, IP; AM; EF), supervised by the first and senior author. The screening process is presented in Fig 1.

**Table 2:** Search strategy.

Concept	Definition	Terms
Mental health	Mental health conditions identified as priority conditions by WHO MH Gap (Version 2.0), which are targeted by country policies in the Global South.	TI (depression OR schizophreni* OR “psychotic disorder*” OR psychos?s OR PTSD OR “posttraumatic stress” OR “post-traumatic stress” OR “psychological trauma*” OR “stress disorder*” OR “mental stress” OR “substance-related disorder*” OR suicide OR “self-harm” OR traumatic OR (mental ADJ2 (health OR ill* OR disorder*)) OR ((alcohol OR drug OR substance) ADJ2 disorder*)) OR AB (depression OR schizophreni* OR “psychotic disorder*” OR psychos?s OR PTSD OR “posttraumatic stress” OR “post-traumatic stress” OR “psychological trauma*” OR “stress disorder*” OR “mental stress” OR “substance-related disorder*” OR suicide OR “self-harm” OR traumatic OR (mental ADJ2 (health OR ill* OR disorder*)) OR ((alcohol OR drug OR substance) ADJ2 disorder*)) OR ID (depression OR schizophreni* OR “psychotic disorder*” OR psychos?s OR PTSD OR “posttraumatic stress” OR “post-traumatic stress” OR “psychological trauma*” OR “stress disorder*” OR “mental stress” OR “substance-related disorder*” OR suicide OR “self-harm” OR traumatic OR (mental ADJ2 (health OR ill* OR disorder*)) OR ((alcohol OR drug OR substance) ADJ2 disorder*)) OR MH (“Mental Health” OR “Mental Disorders” OR “Schizophrenia” OR “Psychotic Disorders” OR “Post-Traumatic Stress Disorder” OR “Self Harm” OR “Depression” OR “Suicide” OR “Substance Abuse” OR “Substance-Related Disorders” OR “Psychological Trauma”)
Social interventions	Interventions that have the capacity to improve the social outcomes of patients and communities (Wahlbeck et, 2017)	((social or family parenting or household or individual or skills or employment or community or NGO* or non-governmental organisation* or school or combination) adj2 (intervention* or program*)). OR ((employment or community or neighbourhood or housing or social) adj2 support*) OR ‘cash transfer’ or grant* OR ‘social support’ OR ‘public policy’ OR ‘peer group’ OR poverty OR social inequality OR stigma*

Before screening began, 30 studies were randomly selected and reviewed by all screeners. We compared individual screening decisions and discussed discrepancies to ensure consistency and reliability across the team before starting the main screening phase, as is recommended for best practice [40]. The first-round screening of titles and abstracts excluded duplicates and studies falling outside the inclusion criteria. Full text screening was preceded by a similar phase of validation. To complement the database search, we manually screened reference lists of eligible studies and performed targeted searches in Google Scholar using the same key terms applied in the primary search. Backward and forwards citation searches were needed given that the terminology relating to ‘social interventions’ is sometimes inconsistent and therefore may miss potentially relevant studies in keyword-based database searches [41]. This process identified an additional 11 studies included in the final dataset.

### Inclusion criteria

Our inclusion criteria for mental health conditions was informed by the mhGAP version 2.0 priority conditions [42], focusing on depression, schizophrenia, suicide, substance-use disorders, and child mental health conditions. We did not include dementia or epilepsy in our review, seeking to focus on mental health conditions that currently contributed to the greatest burden of disease.

We did not set any date limits. Geographical limits were set to low- and middle-income countries, as per the World Bank classification. We included peer-reviewed studies published between 2012–2025 and limited our inclusion to studies published in English. We excluded studies based on secondary data, pilot studies or those that did not explore the intervention impact on mental health outcomes (i.e., cost-effectiveness analyses; meta-analyses).

We also included studies that focused around the general category of mental health, if they were assessed by standardised scales, as this aligns with the ethos of social interventions that are concerned with wellbeing at multiple levels rather than being limited to clinical settings [38,43].

### Data extraction and analysis

Data was extracted using a customised extraction table in Excel. Details included study design, description of intervention, aims of the study, intended beneficiaries, type of social intervention, and level of operation. A separate extraction table for the meta-analysis was created, including country, geographical region, population, sample size, outcome measure, and results. Our data extraction table can be found infile (See S1 Data)

### Quality assessment

Quality appraisal was performed on eligible papers using Joana Briggs critical appraisal tools [44], which is available in the supporting information files (see S2 Data). We did not exclude any papers based on quality.

### Meta-analysis

We completed a meta-analysis based on the most commonly occurring mental health condition in our sample (depression). Following established meta-analytic procedures [45,46], we first calculated study-level effect sizes for depression. For each included study, the standardized mean difference (SMD) was estimated using group means, standard deviation, and sample sizes for intervention and control groups. Effect sizes were expressed as Cohen’s *d* for the depressive symptoms [47]. The pooled effect of the interventions on depression was then estimated using STATA 17.0. STATA provides a z-test in its meta-analysis output, which was used to determine whether the aggregated effect size significantly differed from zero. A statistically significant z-test (P < .05) indicated that interventions had a meaningful impact on depression across studies.

To evaluate variability across studies, we assessed heterogeneity using the I² statistic and the Cochran’s Q-statistic [48]. I² values of 25%, 50%, and 75% were interpreted as low, moderate, and high heterogeneity, respectively [49]. Given that our analyses evidenced high heterogeneity, we analysed the results using random effects models. A significant Q-statistic (P < .001) further indicated that the observed variation in effect sizes was greater than zero.

## Results

**Fig 2:**
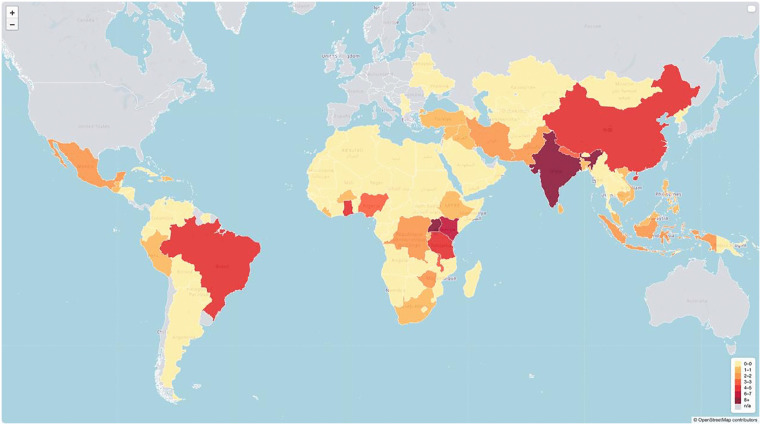
Geographical distribution and frequency of included studies. Base map and data from OpenStreetMap and OpenStreetMap Foundation. © OpenStreetMap Base map tiles © OpenStreetMap contributors (https://www.openstreetmap.org), ODbL license (https://www.openstreetmap.org/copyright). Country border shapefiles from datasets/geo-countries (https://github.com/datasets/geo-countries), derived from Natural Earth public domain data (https://www.naturalearthdata.com/about/terms-of-use/).

We identified 83 studies examining social interventions for mental health across low- and middle-income settings, with insights from 36 countries. The regional representation consisted of 38 studies from Africa, 14 from South Asia, 11 from Latin America and Caribbean, 10 from East Asia and Pacific, and eight from the Middle East. The countries with the most studies were Uganda (n = 8), India (n = 8), and Kenya (n = 6). Geographical distribution of studies is summarised in Fig 2, which indicates the geographical location of identified studies and the number of interventions in each country. As we high income countries were excluded from our criteria, they are labelled as N/A, and coloured in grey.

Almost all studies focused on common mental disorders (CMD), with only 6.1% (n = 5) of studies focusing on serious mental disorders (SMD), all of which centred on schizophrenia. The most common condition of focus was depression, which made up 69.5% (n = 57) of the studies, followed by 8.5% (n = 7) on general mental health, 7.5% (n = 6) on child mental health, 6.1% (n = 5) on schizophrenia, 4.9% (n = 4) on suicide and 4.9% (n = 4) on substance use. In terms of demography, 69 studies were targeted at all genders, while 13 studies were specifically targeted at women. 39 studies were specifically aimed at young people, 37 at adults, five were aimed at families specifically (including young people and adults), and one study included all.

91.5% (n = 75) of identified studies were quantitative. 6.1% (n = 5) studies used mixed methods. Similarly, only 2.4% (n = 2) of the studies were qualitative in nature. Randomised control trials (RCT) were the most common methodological design, making up 67.1% (n = 55) of the studies. Regarding the remaining quantitative studies, eight were quasi-experimental, four were non-randomised, three were cohort studies, and one was a case control study.

### How effective are social interventions? Meta-Analysis Results on depression

Due to the diversity of the mental health conditions of focus and the heterogeneity of measures used to assess effectiveness, we could not conclude a meta-analysis for all papers in our final sample. Our meta-analysis began with 53 studies that examined social interventions on depressive symptoms. Of these, 14 papers provided sufficient statistical information (e.g., means, standard deviations, sample sizes) to be considered for quantitative synthesis. A further 39 were excluded due to inconsistencies in outcome measurement across studies and a lack of statistical details.

**Fig 3:**
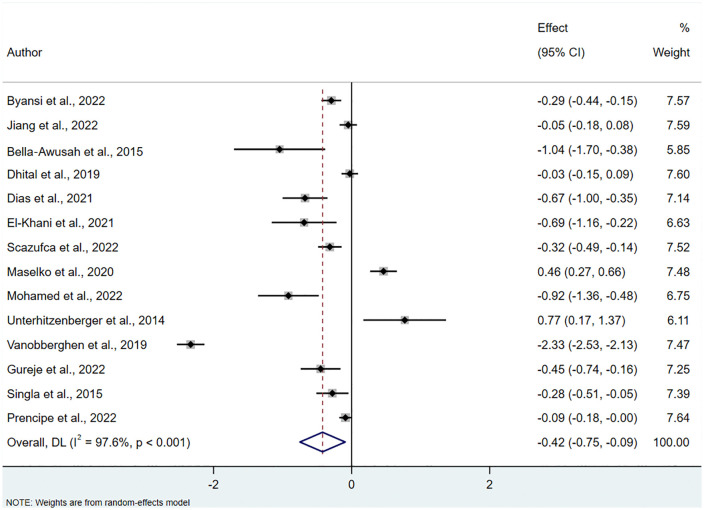
Pooled effect sizes of social interventions for depression in LMICs.

14 studies reported data that allowed for the calculation of standardized mean differences. Using a common outcome of depression, the pooled analysis demonstrated a significant medium effect size (Cohen’s *d* = 0.42; Effect size = -0.42, 95% CI = -0.75 to -0.09, p = 0.005), indicating that social interventions were associated with a meaningful reduction in depressive symptoms. However, heterogeneity across studies was high (I² = 97.6% and Cochran’s Q = 546.12 (df = 13, p < 0.001), suggesting substantial variability in effect sizes. Fig 3 presents the forest plot summarizing the pooled effect sizes for depression across the included studies.

As indicated in the forest plot, effect sizes across individual studies varied widely, ranging from strong negative effects (e.g., [50]) to positive or null results (e.g., [51]). This wide variability highlights differences in intervention effects across settings and study designs. Taken together, these findings suggest that interventions are associated with a moderate reduction in depressive symptoms overall. However, the extremely high heterogeneity warrants caution in interpretation and indicates a need for further analyses to explore potential moderators such as study setting, intervention type, or population characteristics.

### How are social interventions used to impact mental health? Findings from the full dataset

We present our findings responding to our first research question, in line with the framework used to structure our search, outlined previously. It is worth noting however, that due to the nature of social interventions, many were multi-dimensional, and engaged with multiple levels of action. We organised our analysis based on the primary level of delivery described by authors themselves, to provide clarity in discussions of findings.

#### Individual and family level.

We identified six studies discussing social interventions delivered at the individual and family level. Five of the six studies at this intervention level were deemed effective by the respective authors. One intervention found no evidence of effectiveness [52]. Three studies focused on parenting skills and support, all targeting depression outcomes amongst parents. In Ghana, pregnant adolescents were paired with ‘model mothers’, part of the community, to support their transition into motherhood [52]. In Nigeria, a similar model of a ‘neighbourhood mother’ was used to support pregnant mothers with parenting skills, social support, behavioural activation and problem-solving [53]. In China, parents in poor rural areas received parenting skills training, as well as weekly group reading & play activities alongside access to free play space at the parenting centres [54].

Three interventions catered for individuals with specific social needs. In Iraq, an intervention consisted of a psychotherapy programme that incorporated skills training, social empowerment and strength-building for survivors of torture [55]. Another programme delivered in the Democratic Republic of Congo, *Healing in Harmony,* offered survivors of sexual violence and violence-related trauma music therapy, promoting cognitive restructuring and verbalisation of trauma, with the goal of positively changing how recipients engage with their social environment about their experiences [56]. In India, a lay counsellor intervention for older adults combined problem-solving therapy with support for accessing government-sponsored medical services and social support [57].

#### Households and working life level.

Nine studies explored interventions delivered at the household and working life levels. All the nine interventions were deemed to be effective. Five of the papers were produced by a research group researching family-based economic strengthening interventions in Uganda [58–62]. The studies looked at different iterations and implementations of this type of social intervention. Given the signification impact of HIV/AIDS in Uganda, the various studies had various target populations, included AIDS orphaned children, young girls living with HIV or HIV prevention amongst young people. Outcomes were intended to improve mental health of young people, but interventions included their households. For example, families were encouraged to open savings account for the children (Child Development Accounts) which would be matched by the project, caregivers and children were invited to family financial management and microenterprise workshops, as well as mentorship programmes. This suggests the need for intervention elements directly targeted at children, as opposed to general household-targeted interventions, in order to adequately address child mental health.

The other three papers exploring delivery at this level consisted of employment interventions for Malaysian manufacturing workers, directly engaging with socio-structural sources of distress, namely their work environment. In Ghana, as a response to professional and personal challenges that were found to negatively impact teacher mental health, a teacher training intervention was undertaken to strengthen their belonging in the school community [63]. In the Dominican Republic, a living wage intervention for apparel factory workers saw signification reductions in depression levels [64]. The *Small Farmers Development Program (SFDP)* in Nepal included agricultural loans and strengthening of social support structures which supported low-income farmers [65].

#### Community level.

We found 19 papers delivered at community-level interventions. 14 of those interventions were found to be effective, while four of them found no evidence of effectiveness [55,66–68]. One intervention in China found mixed evidence, as there was some effectiveness in terms of reduction of depressive symptoms, but this differed across gender and was not sustained over time [54]. These interventions were typically delivered by or with community members themselves, such as lay community health workers, peers or community volunteers. After initial training, community-members were placed in charge of delivering various intervention elements, often targeting both socio-structural and socio-relational aspects.

For example, in their study on a community psychosocial intervention called *Atmiyata* in Gujarat, India, Pathare and colleagues [69] describe various intervention elements which speak to either socio-structural or socio-relational dimensions. While facilitation to help recipients access social benefits addresses socio-structural dimensions of ill mental health, their broader community awareness elements of the intervention address socio-relational dimensions. This community awareness included engagement with broader community members, during short films screenings, exploring mental health, in social and economic context, linking these to experiences around unemployment, domestic violence or family conflict. *Atmiyata* includes problem-solving mechanisms too, which were also observed in other interventions.

Broström and colleagues [70] assessed a *Youth Friendship Bench* project in Zimbabwe, directed at adolescents experiencing depression, and particularly tailored to address problems relevant to them including sexuality, relationships and young parenthood.

Many interventions delivered at the community-level were peer-led, drawing on peer support mechanisms. In Ghana, Appiah and colleagues (2020) attributed the significant outcomes of the community-based multicomponent positive psychology intervention in part to the effect of the peer delivery of the intervention, indicating the positive social impacts of such interventions delivered at community rather than individual level. Adolescent mothers in an intervention in Malawi similarly included a peer support element, consisting of teenage mothers support groups, alongside other offerings such as health services and child monitoring [71]. In Pakistan, a longer-term peer-led psychosocial intervention consisting of problem-solving and behavioural activation for pregnant women with depression was delivered by other married women in the community [66].

Other interventions focused on creating sustainable support structures and systems within communities, which have the potential of addressing certain root causes of ill mental health and distress, in particular structural ones. In a Kenyan intervention named *Kuja Pamoja* (translating to ‘come together’) described by Goodman and colleagues [72], intervention elements included savings- and lending-groups, as well as skills and entrepreneurial training for women, in an effort address economic challenges and inequalities as drivers of adverse mental health outcomes. In Haiti, given the high likelihood of natural hazards, a disaster preparedness intervention was aimed at supporting mental health of recipients and ensuring they have necessary social support structures in place in case of a disaster [73].

#### Service level.

Social interventions delivered at the level of services, were the most commonly identified in our search, with a total of 31 studies. Amongst these studies, 19 focused on school-based interventions, which we present in the subsequent sub-section. Here we focus on the 12 service-level interventions. 11 out of the 12 non-school-based service-level interventions were found to be effective, and one intervention found no evidence of effectiveness [74].

Five studies delivered at service level targeted individualised offering blended therapeutic interventions with social components. In a comparative study in India and Pakistan, the peer-delivered adaptation of the *Thinking Health Programme* offered psychosocial support to pregnant women experiencing perinatal depression [75]. In Brazil, a task-shared and collaborative programme was delivered for older adults living in socio-economically deprived areas experiencing depression [76]. In Bangladesh, the intervention supplemented regular paediatric visits at the community clinics with psychosocial stimulation for mothers and food supplementation for their children, aimed at improving mothers’ depression [74]. In Indonesia, outpatients with schizophrenia received psychoeducation and social skills interventions in the community [77]. Overall, these interventions incorporated mechanisms such as problem-solving, behavioural activation, social support and psychoeducation in ways that engaged with the social dimensions of people’s distress and helped them devise strategies to address these.

Another two studies, both from Uganda, implemented group-based psychotherapy interventions, which similarly incorporated mechanisms addressing the social aspects of mental health. In an intervention aimed at perinatal depression, women were supported in group-settings to identify and name problems in their day to day life contributing to their distress, and encouraged to collectively support each other in identifying achievable solutions [78]. The other intervention was targeted at people with HIV/AIDS, and included sessions focusing on income-generating skills, to attain livelihood opportunities that foster dignity and independence [79]. Beyond the practical aspects of such interventions geared at providing solutions to socio-structural challenges, participants were encouraged to create groups for sustaining group-based livelihood generating enterprises (for example, growing and selling produce) that incorporates socio-relational elements to these solutions.

Two studies, from India and Brazil, looked at supported housing initiatives aimed to facilitate post-hospitalisation discharge and aid social reintegration into the community. In Brazil, the intervention supported transitions by offering housing and employment support to participants [80]. In India, the intervention focused on shifting paradigms of care towards ones that emphasize independence of people with severe mental health conditions, by promoting community-grounded care [81]. A similar study in Iran, provided intervention focused on community rehabilitation, though it did not include housing, focusing social function for people with chronic mental health concerns [82].

#### School level.

In Wahlbeck’s [38] framework, school-based interventions fall under the service-level category. School-based interventions comprised a quarter of all interventions (n = 19) we identified. The age ranges of young people included in school-based interventions varied, starting from as young as 8 years old to 19 years old. 11 studies were found to be effective and four interventions found no evidence of effectiveness in respect to mental health outcomes of interest in this review [51,83–85]. A further four interventions showed mixed outcomes, indicating some level of improvement on some indicators of interest but not all [86–89]. For example, the intervention by Qouta and colleagues [86], focusing on children in Palestine did not show overall effectiveness, but rather gender- and risk-specific effectiveness, given the complexity of the context within which the intervention was delivered.

Ten interventions were modelled after universal school interventions, aimed at supporting students to identify and work through their problems. In Tanzania, an intervention centred around promoting pro-social behaviours, supporting socio-relational dimensions of mental health for students in various social locations, including school and community [90]. Another intervention in India, the *SEHER* intervention, consisted of multiple components aimed at supporting students in actively engaging with their social problems, including setting up school-wide students committees listening, supporting and engaging concerns; peer-based support groups; and problem-solving counselling sessions with individual students [91]. *RECAP-VN* in Vietnam focused on social skills training in school [92]. In Nigeria, a school-based intervention trained teachers and school nurses on psycho-social wellbeing, with a focus on resiliency, and established peer support groups in order to create environments where students’ social worlds could be better understood, creating opportunities for them to more easily work through their problems [93]. Also in Nigeria, Bella-Awusah and colleagues [50] studied a school-based cognitive behavioural therapy (CBT) programme, which blended religious elements to better make sense of the social anchors in student’s lives.

Other studies (n = 9) were structured around preventing and addressing negative mental health outcomes that linked to specific contextual realities shaped by socio-structural factors. In Guatemala, Kulis and colleagues [94] highlight the particular social, political and economic context in the country that contribute to the vulnerability of young people in regard to substance abuse. As such, the intervention responded to these realities, by emphasising substance use prevention [94]. Similar interventions were observed in Brazil [88,89]. Two interventions in Gaza were aimed at Palestinian children who had lived through conflict and war, focusing on social relationships, peer protection, socio-emotional skills and cooperation with different social actors including schools and families [83,86]. A similar intervention was delivered to war-affected children in Burundi, which blended creative and artistic aspects [87]. In Nepal, school children in low-income settings received teacher-delivered psychosocial interventions focused around mental health and hope in earthquake affected districts [85]. In Rwanda, unstructured emotional writing interventions were delivered to orphan adolescents [51].

#### Country level.

We identified 13 studies reporting on social interventions delivered at country-level, all of which were financial in nature. Out of the 13 studies, 10 interventions were found to be effective and two interventions showed no evidence of effectiveness [95,96]. One intervention in Tanzania found mixed evidence in terms of outcomes, as depressive symptoms decreased amongst men but increased for women [97]. The majority of interventions were cash transfers, of which six were unconditional, three were conditional and two were hybrid. These cash transfers were sometimes delivered in tandem with other intervention elements, for example in Uganda the intervention included business training skills [95,96]. In Tanzania, the Adolescent Cash Plus intervention (which targeted adolescents) included similar livelihood and financial skills training, but also included linkages to health services and supported aspects socially relevant aspects for adolescence including sexual and reproductive health, as authors highlight the importance of multisectoral approaches to supporting the complex needs of adolescent mental health [98,99]. In Kenya, the intervention also included free health insurance alongside the unconditional cash transfer [100].

Eight studies investigated the impact of larger national-scale government-run cash transfer programmes on mental health outcomes specifically. Machado and colleagues [101] investigated the outcomes of Brazil’s programme *Bolsa Familia,* which they found to be associated with 56% lower suicide rates after adjusting for other variables. In India, *Janani Suraksha Yojana (JSY)* is a safe motherhood intervention aimed at supporting poor women, and involves cash assistance upon delivery and post-delivery care. The study found up to 36% reduction in moderate depression amongst recipient mothers in Uttar Pradesh, one of the most deprived states in the country [102,103]. In Malawi, the national unconditional cash transfer programme *Social Cash Transfer Program (SCTP)* directed at ultra-poor and labour-constrained household too was found to have positive effects on youth mental health, particularly amongst females [104]. Authors in particular highlight socio-relational mechanisms contributing to positive outcomes, such as increased sense of social support facilitated through greater opportunities for socialisation and decreased caregiver stress at the household level. Two studies also evaluated Kenya’s Cash Transfer for Orphan and Vulnerable Children (CT-OVC) governmental programme, found to not only considerably decrease depressive symptoms but also improve hope and outlook on future [104,105], similarly highlighting that the ability to meet basic needs opens up opportunities to strengthen socio-relational dynamics that support wellbeing.

Cash transfer interventions normally seek to address poverty, which we found to be the case for all cash transfer interventions included in our sample (n = 11). However, nine of these interventions were also explicit in expanding their efforts to address complex forms of social marginalisation and inequality. For example, four studies focused specifically on interventions targeted at orphaned and vulnerable children (OVA). A study by Haushofer and colleagues [100] directed their health insurance and cash transfer intervention towards informal workers in Nairobi, recognising the increased social and economic vulnerabilities experienced by informal sector workers. Angeles and colleagues [104] highlight the vulnerabilities linked to chronic illnesses and disabilities that is a reality for many of the beneficiaries of their intervention. In Syria, Falb and colleagues [96] highlighted the complex crisis context - involving civil conflict and ISIS occupation – experienced by the intended beneficiaries of the intervention. In their contextual grounding and attention to complex and intersectional forms of disadvantage, these cash transfer interventions highlight the importance of considering the interplay of socio-structural dimensions in relation to mental health.

#### Mixed levels.

Four studies were more distinctively ‘mixed’ in nature in terms of their level of delivery, which we describe here. All four interventions were found to be effective. In Mexico, Amador Buenabad and colleagues [106] studied a multicomponent programme at combined two streams of intervention: *Leaving Traces on Your Life (Dejando Huellitas en tu Vida)* and *Raising Children with Love: Promoting Harmony and Self-Improvement in Mexico (CAPAS-Mx).* The former delivered the intervention to children at school-level, focusing on social and emotional skills, while the latter delivered support to parents at the family level, focusing on parenting skills. An intervention aimed at Syrian refugees living in Lebanon similarly blended school and family level, by offering school-based psychosocial recovery intervention to children and parenting programmes at the family level [107]. Another study in Mexico integrated a life skills education programme delivered at the school-level, with a community-level youth patrol imitative [108]. In Burkina Faso, economic strengthening programmes for delivered to women at community level, which included livelihood training, savings group formations and seed-capital grants, was paired with family coaching sessions delivered at household level to family members [109].

## Discussion

Our findings revealed a diversity of social interventions being used in low- and middle-income settings that, when used independently or in combination, had positive impacts on mental health outcomes. We identified a large amount of heterogeneity in the field, highlighting a diverse range of approaches to addressing social and structural determinants of poor mental health in LMIC settings. While all but four interventions identified had a primary level of delivery which we mapped across Whalbeck’s [38] categories, many also had some elements of heterogeneity: seeking to tackle numerous the social conditions linked to poor mental health in these settings. This finding is of particular relevance as it speaks to the power of social interventions in holding the complexity of people’s lives.

Our attempt to assess the effectiveness of interventions using a meta-analysis was limited by the wide heterogeneity in measures used to evaluate mental health outcomes. This finding reflects a broader challenge linked to capturing the true impact of social interventions. Heterogeneity is an expected outcome given the complexity of social landscapes targeted by these interventions. However, heterogenity of action does not negate the existence of shared conceptual principles that shape wellbeing. An absence of applying conceptual frameworks to the design and evaluation of social interventions causes challenges in measurement of impact, and points to the need for developing theory within the social intervention landscape. For example, recent work seeking to identify active ingredients in social prescribing in the UK had similar challenges in identifying a shared set of principles underpinning efficacy [110].

With better understandings of which types of social dynamics are targeted by specific interventions, studies could apply outcome measures in ways that lend themselves to generating shared understandings, and better understandings of complex impact. For example, condition specific measures of depression may capture different aspects of illness (i.e., social relationships, isolation, cognitive symptoms, physiological symptoms), but we struggle to identify which domains are engaged by approaches. By identifying the ‘active ingredients’ of social interventions such as improving social support, social relations, reducing stigma, or enhancing coping skills, repair and restoration of hope, or collective efficacy, would allow future evaluations to focus on the most relevant outcomes.

The importance of refining our object of measurement in GMH is underscored by funders who are increasingly requesting proposals that outline theories of change and suspected mechanisms that will be measured. This presents a significant challenge to those wanting to include social interventions in their work but lack the acceptable evidence to articulate their value, or impact to funders. Future research clarifying mechanisms in social intervention landscapes is crucial, and must engage with local definitions of wellbeing, and impact within such investigations. This will resist tendencies to build frameworks anchored exclusively to western perspectives of knowledge that minimise cross-cultural differences and variation. Some existing frameworks may offer good starting points to structure and evaluate social interventions more widely, without losing meaning cross-culturally. For example, Corey Keyes’ [111] well-established framework of social wellbeing identifies five domains that have been linked to a dual continuum model of mental health, which encapsulates social wellbeing and full mental health [112]: Social integration, social contribution, social actualisation, social acceptance and social adherence, which has been argued as critical to global mental health practice previously [113]. However, attempts to create frameworks of active ingredients need to be co-produced within culturally and structurally sensitive paradigms that hold complexity [33]as the true value of social interventions, lies in its ability to respond to the specific challenges of a social environment – which demands a hyper-local perspective on scale and impact, which counters current priorities.

We also note that assessing impact of social interventions was largely reliant on quantitative methods. This itself may also shape the limited clarity on the of ‘active ingredients’ within social interventions. Despite the use of wide inclusion criteria which included the use of qualitative studies, our review only identified two studies using qualitative methods to describe the impact of interventions. Developing understandings of social processes is more aligned with qualitative evaluations, which we suggest should be expanded in this field, aligning with earlier calls for Global Mental Health research evaluate the methodological relevance of the tools we use to address these dimensions [114,115].

While quantitative research design yield important insights, there are necessary considerations around context, and lived experience that these may not be able to capture. Qualitative studies will be important to inform the future of social interventions, as these can help us better articulate the why and how these interventions work. Some qualitative methods relevant to the study of social interventions include ethnographic studies, which have been highlighted for their ability to centre localised conceptualisation of (good) mental health drawing on context-specific historical, social, and political realities [1]. Overall, target communities were usually not involved in the conceptualisation and design phases of social interventions. This perhaps points to an oversight of local voices, as focus is on scalability over specificity (as is indicative in the dominance of randomised-control trial design). Participatory methods may be of significant value, particularly in their ability to include and centre community voices in the conceptualisation and design of relevant interventions.

The dominance of child- and youth-focused social interventions for mental health in our settings, reflects a strong recognition of the importance of early interventions for mental health, and a clear response to the growing burden of mental health conditions among young populations [116–118]. Schools, as structured and accessible settings, provide a natural entry point for the design and delivery of such interventions, making them both feasible and scalable [119–121]. Yet, this convenience also raises concerns about the risk of these programs becoming institutionalized “tick-box exercises” that prioritize coverage over depth, potentially undermining their effectiveness and long-term impact. Furthermore, the reliance on school-based platforms highlights a gap in reaching individuals outside the formal education systems, such as out-of-school youth, marginalized children, or those in unstable living conditions. These groups may have equal, if not greater, need for psychosocial support, yet remain harder to access and often excluded from intervention efforts. Future research and practice must therefore balance the advantages of structured delivery within schools with innovative strategies to engage populations that fall outside these systems.

Overall, our findings indicate that the levels of engagement with social interventions were flexible, and rarely neatly fit rigidly pre-determined categories. As such, social interventions observed in this review were boundary spanning, allowing movement between individual, community and society levels of action. There is an immense, and under appreciate value to the ability of these types of interventions to hold complexity and produce true ‘ecosystem’ type approaches to addressing mental health challenges. For example, the *Atmiyata* intervention in India offered psychosocial interventions delivered by community volunteers but equally considered the importance of opening up community-level discussions around social issues impacting mental health, such as unemployment, domestic violence, family conflict and alcoholism [69]. This recognises care not only as an individual-level offering, but as an effort to build environments that have the capacity to be caring. In Bangladesh, the intervention for maternal depression not only offered clinical care, as might often be the case for such interventions, but also provided food supplementation for children [74], recognising that much of the mental health of mothers is bound up in material demands that exist outside of the clinic. Such approaches align with recent calls within the WHO for mental health in all government sectors [122].

Our findings potentially direct us towards the value of different logics of care in mental health landscapes, which more actively acknowledges the numerous social aspects of care needed for improved mental health. Elsewhere, we have described this as the need for logics of care that centre remedy and repair; where the objective of action is to also ensure that we are working to build *mental health sustaining environments* [123,124]. Such logics of care demand that we expand our typical remit of engagement with social dynamics, to include action at political and structural dynamics of everyday life. The object of care would be to support others in the process of building ‘better worlds’ and ‘repairing’ the social fabrics and broken social environments that lead to the expression and development of poor mental health, rather than force complicated demands for justice to fit into psychological language which flattens need The social interventions described in this review acknowledge that mental health related repair can be achieved through action in small scale action in the social landscapes of peoples lives at the levels of individuals, communities, as well as populations..

Notwithstanding, this review has several limitations that should be considered when interpreting the findings. First, the included studies examined a wide range of outcomes and employed diverse measures. Because of this variability, we were only able to conduct a meta-analysis on depression outcomes. This restricted scope reduces the ability to capture more nuanced patterns of causation or correlation that might exist across different outcomes in individual studies. Second, the availability of data across studies was limited. Many studies did not report sufficient short-term or long-term follow-up data, making it difficult to draw firm conclusions about the sustainability of intervention effects over time. Third, although our inclusion criteria focus on studies with experimental or controlled conditions to strengthen the rigor of the synthesized evidence, this decision also reduced the number of studies that could be included in the review. As a result, potentially relevant evidence from other study designs was not captured. We also focused on a sub-set of conditions from the mhGAP (2^nd^ version), meaning that other conditions may link to sets of other social interventions within the literature may not have been captured here. We suggest that future studies build on our findings, organising searches around the recent update of the mhGAP, launched after the start of our review, or explore alternative sub-categories of interest (for example, mental health conditions in old age, such as Dementia). Finally, our search strategy relied primarily on English-language databases and search terms. This approach may have excluded relevant studies published in languages other than English or indexed on non-English databases, which could have provided additional insights and strengthened the comprehensiveness of the review.

## Conclusion

The importance of addressing social determinants in the Global Mental Health field is widely acknowledged [125]. Yet the uptake of social interventions does not align with anticipated demands. For example, the scale of social development needs in LMIC settings is immense. Across countries, many communities face challenges in access to basic social welfare needs – such as safe housing, WASH infrastructure. Access to employment opportunities remain unequally distributed. For example, many of the countries most represented in our review have high GINI-coefficients, indicating particularly high levels of income inequality [126,127]. Social interventions in these landscapes matter more than ever – as people living with mental illness are known to face multiple intersecting forms of oppression that decrease their access to social welfare supports and needs [33,37].

Our work suggests that a range of effective interventions that take seriously calls for action and attention to the unequal social worlds where mental health is lived, ***do exist***. As such, we take Wahlbek’s [38] crucial point – that lack of evidence does not equal lack of effectiveness – as a rallying cry – to expand our focus on social interventions, with calls for further discovery research into this area.

Future work should focus on the value of social interventions such as social enterprise and employment training as embedded into mental health supports in LMIC settings, or interventions creating opportunity for social justice as this is the area we found the least evidence to date. Finally, while the list of social interventions outlined in Whalbeck’s work served as a helpful orientation for our review, and a good example of the range of social interventions available, this cannot serve as a fixed category for exploration. The power of social interventions is that they will vary depending on context. As such, rather than argue or benchmark ‘gold standard’ social intervention types in LMICs, or compare them against those developed in HICs, we urge future studies to explore and interrogate the field with diversity and curiosity as their starting point.

Social interventions are as expansive as the social worlds we live in. Opening ourselves up to these diversities and opportunities, often embedded in community -led responses to mental health challenges [128,129], remains an un-tapped opportunity for our field.

Further work in this area will illuminate the undeniable truth that efforts to improve mental health, must be inseparable from investments to strengthen the social fabrics of our lives, and are an equally valued investment for worlds where mental health for all is finally a reality.

## Supporting information

S1 Checklist PRISMA checklist. (DOCX)

S1 Data Data extraction file. (XLSX)

S2 Data Quality assessment checklist. (XLSX)

S1 Table Meta analysis extraction table. (XLSX)
