# Supplementary material for: The power of the social: A systematic review and meta-analysis of social interventions in Global Mental Health
Source: PLOS Ment Health. 2026 Jul 1;3(7):e0000625. doi: 10.1371/journal.pmen.0000625 (PMC13322564; doi:10.1371/journal.pmen.0000625)
Supplement: Attachment — Submitted filename: Response to reviewer_social interventions systematic review.docx [file pmen.0000625.s005.docx]

*The power of the social: A systematic review and meta-analysis of social interventions in Global Mental Health.*

Response to reviewer queries

Overall: Thank you for your kind and generous feedback on our review. We have addressed each of your comments in turn, which we think has strengthened the manuscript a great deal. We also have made a range of changes to typos/lack of clarity throughout the text, which we hope increases the readability overall.

Reviewer 1:

| **Reviewer comment** | **Location of change** | **Response to reviewer** |
| --- | --- | --- |
| Concepts and terminology: What is a “GMH context”? If the authors are referring exclusively to LMICs, consistent use of that terminology will add clarity. | p.4 and p.2 | Changed to say ‘across contexts studied in GMH’. We have also clarified what the aims of the field of global mental health are more broadly, which we hope will provide greater context to the drivers behind our study. |
| It could be useful to add a table that lists the main targets of most social interventions in HICs as well as LMICs. This would help readers understand the range of social outcomes these interventions most commonly target in relation to mental health and mental disorders. Some of this is mentioned in the text. A more comprehensive list would also show which types have not been studied in LMICs to date. | p.4 and pg 24 | Thank you for this comment. We have added a description to explain that table 1 contains the most common types of social interventions in HIC found by Wahlbeck et al (2017).  Your comment has also pushed us to articulate the importance of diversity in social interventions – and have also added the following text to the discussion section manuscript:  *While the list of social interventions outlined in Whalbek’s work served has a helpful orientation for our review, and a good example of the range of social interventions available, this cannot serve as a fixed category. Social interventions will and must vary depending on context. As such, rather than argue or benchmark social interventions in LMICs against social interventions in HICs, we urge future studies to explore and interrogate the field with this as a starting point.* |
| Can the authors clarify the mental health conditions included? Grief is noted as a search term in Table 2, but doesn’t appear to be a priority condition in mhGAP 3.0. What guided the exclusion of other categories included in mhGAP 3.0? | p.7 and pg 23 | When we conducted our initial searches in 2022, the mhGap 3.0 was not published (published in 2023). We acknowledge that this is a limitation in our review, and have added this to our discussion section.  When working with the conditions published in version 2.0, we focused on those most occupying the majority of focus in the field: common mental disorders and Severe Mental illness (schizophrenia). This emphasis was also done to make the review more manageable, to allow us to gain a better understanding of how social interventions manifest in these two broad areas. We added the below to the text:  *We did not include dementia or epilepsy in our review, seeking to focus on mental health conditions that currently contributed to the greatest burden of disease.*  Finally, we believe that there was an error in the cut and paste of our search terms. We did not use grief in our search terms. We have re-inserted an example of search terms in the text, which is accurate. Thank you for pointing this out to us. |
| Is there a citation for Joana Briggs critical appraisal tools? | p.8 | Citation was added. |
| Figure 1: There are no numbers associated with “records marked as ineligible by automation tools” or “records removed for other reasons | p.5 | Figure has been updated to include records |
| Recognizing the complexity of some of these interventions, a question arose for me about the meaning of ‘level of intervention’ as I read the results section. I wonder if the authors could refer to ‘level of intervention implementation’ in Table 1. This would distinguish between where/how the intervention is delivered/implemented and the level of outcomes expected. An intervention delivered by a community health worker may have an intended outcome at the individual level, but an intervention delivered by a community-based organization may also have an intended outcome at the level of the community. The language used in the table “levels where social interventions may operate” could be interpreted as implementation level or level of outcome. Similarly, the authors refer to “level of action” in the discussion section, but this also adds to the lack of clarity. | Throughout discussion – see highlighted text | Thank you for this helpful comment. We think that this distinction is an important one, and your comment crystalises what the struggle is for many engaging in this space. That the level of action targeted by an intervention does not always translate to the levels that impact is felt. Our finding which highlights that most interventions were boundary spanning seeks to speak to this point.  However, we have now made changes throughout the manuscript to say *‘the level of delivery”* in our findings. |
| A major challenge of the paper is that the text does not consistently provide information about the effectiveness of the interventions described (outside of those included in the meta-analysis and those presented at the Country Level ). Starting at the Country level, the results section incorporates intervention outcomes following their descriptions (e.g. Bolsa Familia outcomes related to suicide are reported) This would be helpful throughout the paper. This could be as simple as a count of the number of effective interventions at each level, or a tabular summary description of effectiveness for specific outcomes that then refers to the comprehensive table in the appendices. | p. 12, p.13, p.14, p.15., p.16, p.17, p.18 | Thank you for this comment, which we feel will streamline the manuscript for many readers.  For each level, we have added an overview of the effectiveness of all studies at that level. This was added in the first paragraph of level sub-section.  For example, for the community-level we added:  14 of those interventions were found to be effective, while four of them found no evidence of effectiveness (Bass et al, 2016; Maselko et al, 2020; Esmaeilzadeh and Oz, 2020; Miller et al, 2020). One intervention in China found mixed evidence, as there was some effectiveness in terms of reduction of depressive symptoms, but this differed across gender and was not sustained over time (Jiang et al, 2021). |
| What is the difference between “n/a” and “0” in Figure 2 for regions in which studies were conducted (e.g. n/a vs 0 studies in Latin America and Caribbean)? | p.8 | ‘N/A’ pertains to countries that were not included as part of the review (i.e. high income countries). ‘0’ pertains to countries that were included in the review, but for which we found no papers describing social intervention. We have clarified this in the paper. |
| Typo in the reporting of numbers of studies on suicide | p.9 | We have corrected the typo. |
| Please make the conclusions clearer. Can the authors distill what the reader should walk away with? | p. 23 | Thank you for this feedback. We have refined the conclusion, and added a sharper introduction to the discussion overall, to clarify the main take aways from our review. |

Reviewer 2:

Thank you for your strong endorsement of our work.

*No revisions requested*
