# Supplementary material for: The power of the social: A systematic review and meta-analysis of social interventions in Global Mental Health
Source: PLOS Ment Health. 2026 Jul 1;3(7):e0000625. doi: 10.1371/journal.pmen.0000625 (PMC13322564; doi:10.1371/journal.pmen.0000625)
Supplement: Peer review history

# Peer review: Decision Letter 0

**DOI:** 10.1371/journal.pmen.0000625.r001

**Authors:** Karli Montague-Cardoso

10 Mar 2026

PMEN-D-25-00561

The power of the social: A systematic review and meta-analysis of social interventions in Global Mental Health

PLOS Mental Health

Dear Dr. Burgess,

Thank you for submitting your manuscript to PLOS Mental Health. I am very sorry for the delay. After careful consideration of the reviewer reports, we invite you to submit a revised version of the manuscript that addresses the points raised during the review process. You can find all of these comments below. Please feel free to reach out to me directly if you have any questions.

Please submit your revised manuscript by Apr 08 2026 11:59PM. If you will need more time than this to complete your revisions, please reply to this message or contact the journal office at mentalhealth@plos.org. When you're ready to submit your revision, log on to https://www.editorialmanager.com/pmen/ and select the 'Submissions Needing Revision' folder to locate your manuscript file.

Please include the following items when submitting your revised manuscript:

- A letter that responds to each point raised by the editor and reviewer(s). You should upload this letter as a separate file labeled 'Response to Reviewers'.
- A marked-up copy of your manuscript that highlights changes made to the original version. You should upload this as a separate file labeled 'Revised Manuscript with Track Changes'.
- An unmarked version of your revised paper without tracked changes. You should upload this as a separate file labeled 'Manuscript'.

Guidelines for resubmitting your figure files are available below the reviewer comments at the end of this letter.

We look forward to receiving your revised manuscript.

Kind regards,

Dr Karli Montague-Cardoso

Executive Editor

PLOS Mental Health

Journal Requirements:

1. We have noticed that you have uploaded Supporting Information files, but you have not included a list of legends. Please add a full list of legends for your Supporting Information files after the references list.

If the reviewer comments include a recommendation to cite specific previously published works, please review and evaluate these publications to determine whether they are relevant and should be cited. There is no requirement to cite these works unless the editor has indicated otherwise.

Please review your reference list to ensure that it is complete and correct. If you have cited papers that have been retracted, please include the rationale for doing so in the manuscript text, or remove these references and replace them with relevant current references. Any changes to the reference list should be mentioned in the rebuttal letter that accompanies your revised manuscript. If you need to cite a retracted article, indicate the article’s retracted status in the References list and also include a citation and full reference for the retraction notice.

Additional Editor Comments (if provided):

[Note: HTML markup is below. Please do not edit.]

Reviewers' comments:

Reviewer's Responses to Questions

**Comments to the Author**

1. Does this manuscript meet PLOS Mental Health’s publication criteria? Is the manuscript technically sound, and do the data support the conclusions? The manuscript must describe methodologically and ethically rigorous research with conclusions that are appropriately drawn based on the data presented.

Reviewer #1: Yes

Reviewer #2: Yes

**********

2. Has the statistical analysis been performed appropriately and rigorously?

Reviewer #1: I don't know

Reviewer #2: Yes

**********

3. Have the authors made all data underlying the findings in their manuscript fully available (please refer to the Data Availability Statement at the start of the manuscript PDF file)?

The PLOS Data policy requires authors to make all data underlying the findings described in their manuscript fully available without restriction, with rare exception. The data should be provided as part of the manuscript or its supporting information, or deposited to a public repository. For example, in addition to summary statistics, the data points behind means, medians and variance measures should be available. If there are restrictions on publicly sharing data—e.g. participant privacy or use of data from a third party—those must be specified.

Reviewer #1: Yes

Reviewer #2: Yes

**********

4. Is the manuscript presented in an intelligible fashion and written in standard English?

PLOS Mental Health does not copyedit accepted manuscripts, so the language in submitted articles must be clear, correct, and unambiguous. Any typographical or grammatical errors should be corrected at revision, so please note any specific errors here.

Reviewer #1: Yes

Reviewer #2: Yes

**********

5. Review Comments to the Author

Please use the space provided to explain your answers to the questions above. You may also include additional comments for the author, including concerns about dual publication, research ethics, or publication ethics. (Please upload your review as an attachment if it exceeds 20,000 characters)

Reviewer #1: This is an important paper that aims to answer 2 research questions about social interventions in low- and middle-income country settings (LMICs): 1) What types of social interventions exist and how are they used? (i.e., Which populations and key mental health outcomes they target?), and (2) What is the effectiveness of social interventions in reducing mental health symptoms? As the authors note, this is an area of research in which significant evidence gaps persist.

The authors provide a very useful delineation of social relational and social structural interventions. They present a rigorous screening process and supplementary citation searches to ensure capture of social interventions. The discussion section emphasizes the need for conceptual models that would enable developers and evaluators of social interventions to better understand the intervention targets, active ingredients, and to better measure the outcomes of these interventions. Importantly, the authors emphasize the need for attention to culturally and structurally congruent definitions of wellbeing and impact.

Addressing minor and more substantive questions could improve responsiveness to the manuscript’s aims.

Introduction

• Concepts and terminology: What is a “GMH context”? If the authors are referring exclusively to LMICs, consistent use of that terminology will add clarity.

• It could be useful to add a table that lists the main targets of most social interventions in HICs as well as LMICs. This would help readers understand the range of social outcomes these interventions most commonly target in relation to mental health and mental disorders. Some of this is mentioned in the text. A more comprehensive list would also show which types have not been studied in LMICs to date.

Methods:

• Can the authors clarify the mental health conditions included? Grief is noted as a search term in Table 2, but doesn’t appear to be a priority condition in mhGAP 3.0. What guided the exclusion of other categories included in mhGAP 3.0?

• Is there a citation for Joana Briggs critical appraisal tools?

Minor:

• Figure 1: There are no numbers associated with “records marked as ineligible by automation tools” or “records removed for other reasons”

Results:

• Recognizing the complexity of some of these interventions, a question arose for me about the meaning of ‘level of intervention’ as I read the results section. I wonder if the authors could refer to ‘level of intervention implementation’ in Table 1. This would distinguish between where/how the intervention is delivered/implemented and the level of outcomes expected. An intervention delivered by a community health worker may have an intended outcome at the individual level, but an intervention delivered by a community-based organization may also have an intended outcome at the level of the community. The language used in the table “levels where social interventions may operate” could be interpreted as implementation level or level of outcome. Similarly, the authors refer to “level of action” in the discussion section, but this also adds to the lack of clarity.

• A major challenge of the paper is that the text does not consistently provide information about the effectiveness of the interventions described (outside of those included in the meta-analysis and those presented at the Country Level ). Starting at the Country level, the results section incorporates intervention outcomes following their descriptions (e.g. Bolsa Familia outcomes related to suicide are reported) This would be helpful throughout the paper. This could be as simple as a count of the number of effective interventions at each level, or a tabular summary description of effectiveness for specific outcomes that then refers to the comprehensive table in the appendices.

Minor:

• What is the difference between “n/a” and “0” in Figure 2 for regions in which studies were conducted (e.g. n/a vs 0 studies in Latin America and Caribbean)?

• Typo in the reporting of numbers of studies on suicide

Conclusion

• Please make the conclusions clearer. Can the authors distill what the reader should walk away with?

Please do a careful proofreading of the manuscript to correct typos, missing words, etc.

Reviewer #2: Dear Authors,

You did a good job! Your work and findings contribute a lot to the field. The structure of the writing was very well capture every section of the manuscript structure. Supporting literatures and explanation are well addressed. Your work and finding contribute positively to the field.

**********

6. PLOS authors have the option to publish the peer review history of their article (what does this mean?). If published, this will include your full peer review and any attached files.

**Do you want your identity to be public for this peer review?**  If you choose “no”, your identity will remain anonymous but your review may still be made public.

For information about this choice, including consent withdrawal, please see our Privacy Policy.

Reviewer #1: No

Reviewer #2: **Yes:** Assoc. Prof. Dr. Nil Ean

**********

[NOTE: If reviewer comments were submitted as an attachment file, they will be attached to this email and accessible via the submission site. Please log into your account, locate the manuscript record, and check for the action link "View Attachments". If this link does not appear, there are no attachment files.]

-->Figure Resubmissions:

While revising your submission, we strongly recommend that you use PLOS’s NAAS tool (https://ngplosjournals.pagemajik.ai/artanalysis) to test your figure files. NAAS can convert your figure files to the TIFF file type and meet basic requirements (such as print size, resolution), or provide you with a report on issues that do not meet our requirements and that NAAS cannot fix.

After uploading your figures to PLOS’s NAAS tool - https://ngplosjournals.pagemajik.ai/artanalysis, NAAS will process the files provided and display the results in the "Uploaded Files" section of the page as the processing is complete. If the uploaded figures meet our requirements (or NAAS is able to fix the files to meet our requirements), the figure will be marked as "fixed" above. If NAAS is unable to fix the files, a red "failed" label will appear above. When NAAS has confirmed that the figure files meet our requirements, please download the file via the download option, and include these NAAS processed figure files when submitting your revised manuscript.

# Author comment: Author response to Decision Letter 1

**DOI:** 10.1371/journal.pmen.0000625.r002

29 Apr 2026

Attachment Submitted filename: Response to reviewer_social interventions systematic review.docx

# Editor report: Decision Letter 1

**DOI:** 10.1371/journal.pmen.0000625.r003

**Authors:** Karli Montague-Cardoso, Karli Montague-Cardoso

1 May 2026

The power of the social: A systematic review and meta-analysis of social interventions in Global Mental Health

PMEN-D-25-00561R1

Dear Prof. Burgess,

We are pleased to inform you that your manuscript 'The power of the social: A systematic review and meta-analysis of social interventions in Global Mental Health' has been provisionally accepted for publication in PLOS Mental Health.

Before your manuscript can be formally accepted you will need to complete some formatting changes, which you will receive in a follow up email. A member of our team will be in touch with a set of requests.

Please note that your manuscript will not be scheduled for publication until you have made the required changes, so a swift response is appreciated.

IMPORTANT: The editorial review process is now complete. PLOS will only permit corrections to spelling, formatting or significant scientific errors from this point onwards. Requests for major changes, or any which affect the scientific understanding of your work, will cause delays to the publication date of your manuscript.

If your institution or institutions have a press office, please notify them about your upcoming paper to help maximize its impact. If they'll be preparing press materials, please inform our press team as soon as possible -- no later than 48 hours after receiving the formal acceptance. Your manuscript will remain under strict press embargo until 2 pm Eastern Time on the date of publication. For more information, please contact mentalhealth@plos.org.

Thank you again for supporting Open Access publishing; we are looking forward to publishing your work in PLOS Mental Health.

Best regards,

Karli Montague-Cardoso

Staff Editor

PLOS Mental Health

***********************************************************

Reviewer Comments (if any, and for reference):
